# The potential role of brain renin‐angiotensin system in the neuropathology of Parkinson disease: Friend, foe or turncoat?

**DOI:** 10.1111/jcmm.18495

**Published:** 2024-06-20

**Authors:** Zainah Al‐Qahtani, Hayder M. Al‐kuraishy, Ali I. Al‐Gareeb, Ali K. Albuhadily, Naif H. Ali, Athanasios Alexiou, Marios Papadakis, Hebatallah M. Saad, Gaber El‐Saber Batiha

**Affiliations:** ^1^ Neurology Section, Internal Medicine Department, College of Medicine King khaled university Abha Saudi Arabia; ^2^ Clinical pharmacology and medicine, college of medicine Mustansiriyah University Baghdad Iraq; ^3^ Department of Internal Medicine, Medical College Najran University Najran Saudi Arabia; ^4^ University Centre for Research & Development Chandigarh University Mohali India; ^5^ Department of Science and Engineering Novel Global Community Educational Foundation Hebersham New South Wales Australia; ^6^ Department of Research & Development, Funogen Athens Greece; ^7^ Department of Research & Development AFNP Med Wien Austria; ^8^ Department of Surgery II University Hospital Witten‐Herdecke Wuppertal Germany; ^9^ Department of Pathology, Faculty of Veterinary Medicine Matrouh University Matrouh Egypt; ^10^ Department of Pharmacology and Therapeutics, Faculty of Veterinary Medicine Damanhour University Damanhour AlBeheira Egypt

**Keywords:** Parkinson's disease, renin‐angiotensin system

## Abstract

Parkinson disease (PD) is one of the most common neurodegenerative diseases of the brain. Of note, brain renin‐angiotensin system (RAS) is intricate in the PD neuropathology through modulation of oxidative stress, mitochondrial dysfunction and neuroinflammation. Therefore, modulation of brain RAS by angiotensin receptor blockers (ARBs) and angiotensin‐converting enzyme inhibitors (ACEIs) may be effective in reducing the risk and PD neuropathology. It has been shown that all components including the peptides and enzymes of the RAS are present in the different brain areas. Brain RAS plays a critical role in the regulation of memory and cognitive function, and in the controlling of central blood pressure. However, exaggerated brain RAS is implicated in the pathogenesis of different neurodegenerative diseases including PD. Two well‐known pathways of brain RAS are recognized including; the classical pathway which is mainly mediated by AngII/AT1R has detrimental effects. Conversely, the non‐classical pathway which is mostly mediated by ACE2/Ang1‐7/MASR and AngII/AT2R has beneficial effects against PD neuropathology. Exaggerated brain RAS affects the viability of dopaminergic neurons. However, the fundamental mechanism of brain RAS in PD neuropathology was not fully elucidated. Consequently, the purpose of this review is to disclose the mechanistic role of RAS in in the pathogenesis of PD. In addition, we try to revise how the ACEIs and ARBs can be developed for therapeutics in PD.

## 1 INTRODUCTION

**FIGURE 1:**
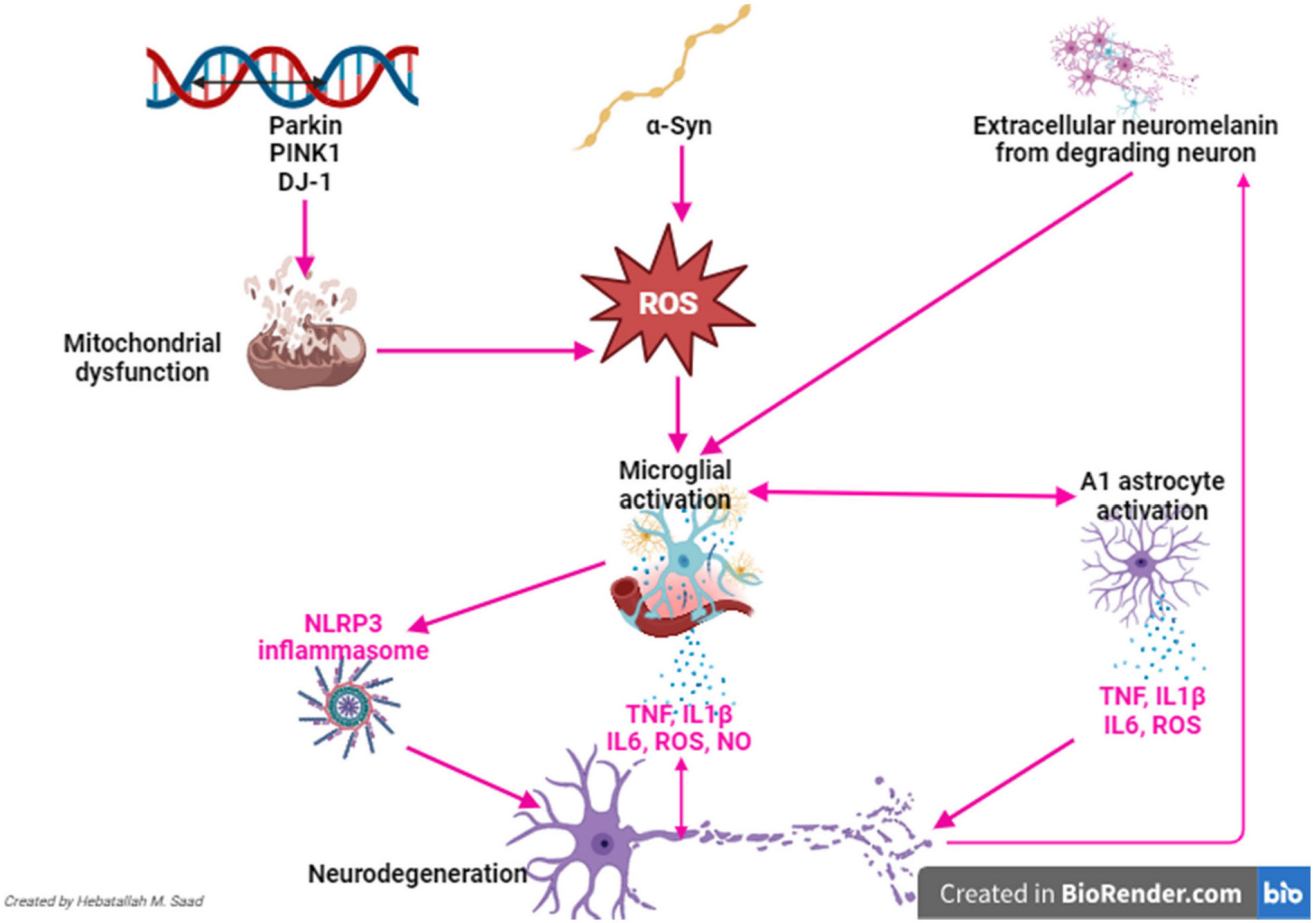
Pathophysiology of PD.

Parkinson disease (PD) is one of most common neurodegenerative disease of the brain.^1^ The pathogenesis of PD is related to the progressive accumulation of mutant and insoluble alpha‐synuclein (α‐Syn) and Lewy bodies in the dopaminergic neurons in the sunstantia nigra (SN).^2^ These neuropathological changes which are mediated by directly effect of α‐Syn and indirectly by α‐Syn‐associated oxidative stress and inflammatory reactions trigger progressive degeneration of the dopaminergic neurons in the SN.^3^ It has been established from preclinical studies that 70% loss of dopaminergic neurons in the SN is established before the development of PD symptoms.^4^, ^5^ Of interest, participation and the presence of α‐Syn in PD neuropathology is debatable could be pathogenic or as a compensatory mechanism to mitigates the loss of the dopaminergic neurons in the SN.^6^ It has been observed that 90% of PD cases are sporadic (idiopathic) though; only 10% PD cases are of familial type which is chiefly related to the α‐Syn neuropathology.^6^, ^7^, ^8^ Interestingly, the interactions between the environmental and genetic factors are highly involved in the pathogenesis of PD.^9^ Thus, the PD is regarded as a heterogeneous neurological disease because of involvement of environmental and genetic factors.^10^ The pathogenesis of PD is very complex and subjected to three temporal phases including triggers, facilitators and aggravators.^11^ The trigger factors such as environmental toxins can induce the facilitators such as systemic inflammations which provoke aggravators such as autophagy dysfunction. These factors are interrelated in the pathogenesis of PD by enhancing the accumulation of α‐Syn and associated oxidative stress, inflammatory reactions, mitochondrial dysfunction and progressive deaths of the dopaminergic neurons in the SN.^12^, ^13^ In PD neuropathology, microglial activation is plays a central role by activating the expression and the release of inflammatory signalling pathway nod‐like receptor pyrin 3 (NLRP3) inflammasome and pro‐inflammatory cytokines such as IL‐1β, IL‐6 and TNF‐α. Besides, the activated microglia triggers the activation of astrocytes which also promote the expression and the release of pro‐inflammatory cytokines.^13^, ^14^ Mutated Parkin, *PINK1* and *DJ‐1* genes that induce mitochondrial dysfunction together with mutant α‐Syn and the extracellular neuromelanin from damaged dopaminergic neurons contribute in the activation of microglia and the development of neurodegeneration in the SN,^12^, ^13^, ^15^, ^16^, ^17^ (Figure 1).

The clinical presentations of PD including; motor features which are rigidity, tremor, bradykinesia and postural instability, and non‐motor features such as sleep disorders, cognitive impairment, dementia, sexual dysfunction and neuropsychiatric disorders. Remarkably, non‐motor features precede the onset of motor features by decades; therefore PD in this prodromal phase may be misdiagnosed with neuropsychiatric diseases.^12^, ^13^ Generally, the incidence of PD is higher in subjects >65 years, it affects 1%–3% of general population, and this percentage is augmented with increasing age.^16^, ^18^ The prevalence of PD seems to be higher in population age >80 years. Conversely, the development of PD <50 years and <21 years is called early‐onset PD and juvenile PD, respectively.^5^

Furthermore, PD neuropathology may be affected by various cardiometabolic diseases such as obesity, hypertension, dyslipidemia and related medications.^16^, ^19^ Different studies indicated that brain renin‐angiotensin system (RAS) may affect the pathogenesis of PD. These claims came from the effects of antihypertensive RAS inhibitors such as angiotensin receptor blockers (ARBs) and angiotensin‐converting enzyme inhibitors (ACEIs) on the PD neuropathology.^20^, ^21^, ^22^ It has been illustrated that ACEIs and ARBs have neuroprotective effects against PD neuropathology.^23^, ^24^ However, the fundamental neuroprotective mechanisms of ACEIs and ARBs are not fully elucidated. Thus, this review aims to clarifying the role of brain RAS in the pathogenesis of PD, and how ACEIs and ARBs mitigate PD neuropathology.

## 2 OVERVIEW OF RAS

RAS is considered as a humoral system concerned with regulating of the electrolytes and systemic blood pressure.^25^, ^26^ The RAS is consisting of various peptides and related enzymes that is diffusely expressed in the most body tissues. The liver angiotensinogen is the main precursor for the synthesis of angiotensin I (AngI) by the action of renin which released from kidney.^27^ The AngI is further converted by angiotensin‐converting enzyme (ACE) to the more active AngII, this pathway is inhibited by ACEIs. However, AngI can be converted by the cathepsin G and chymase, and bypassing the inhibited ACE.^28^ The AngII induces differential effects according to the activated receptors, it produces vasoconstriction and pro‐inflammatory effect via AT1R, and provokes vasodilation and anti‐inflammatory effects via AT2R.^28^ Thus, AT2R counteracts the deleterious effects of AT1R.^29^ Moreover, the RAS can induce paracrine and autocrine effects regardless of its systemic effects.^30^ The harmful effect of AngII such as oxidative stress, mitochondrial dysfunction and inflammation is mediated through activation of NADPH oxidase.^26^ It has been revealed that exaggerated NADPH oxidase activity and associated oxidative stress is linked with the development of age‐related disorders such as PD.^26^ These observations suggest that exaggerated RAS has detrimental effects by inducing oxidative stress and mitochondrial dysfunction, and may implicate in the pathogenesis of PD.

## 3 BRAIN RAS

It has been documented that RAS has more specific role in the brain than peripheral tissues, since; AngII concentration is higher in the brain than peripheral circulation.^31^ Notably, AngII and other components of RAS in the peripheral circulation cannot cross the blood brain barrier (BBB).^31^ Nevertheless, prorenin and angiotensinogen was discovered in the brain,^32^ suggesting a separated and unique brain RAS which differed from that of systemic RAS.^33^ It has been established that brain prorenin promotes the activation of neuronal RAS by cleavage of neuronal angiotensinogen which derived from astrocytes. It been confirmed that 90% of brain angiotensinogen is derived from astrocytes, microglia and neurons.^34^ The brain RAS is involved in the regulation of memory and cognitive function, and in the controlling of central blood pressure. However, exaggerated brain RAS is implicated in the pathogenesis of different neurodegenerative diseases including PD.^33^, ^35^ Indeed, the components of RAS are highly expressed in the nigrostrial pathway and basal ganglion.^36^ Moreover, brain RAS mainly AngII, AT1R, AT2R, ACE2 and ACE are involved in the regulation of dopaminergic neurons in the SN. However, exaggerated of brain RAS is implicated in the degeneration of the dopaminergic neurons in the SN through the induction of inflammation and oxidative stress.^36^, ^37^ Besides, AT1R, AT2R and prorenin receptors are extremely expressed in glial cells mainly in microglial and astrocytes signifying the possible role of RAS in the development of brain inflammatory and oxidative stress disorders.^32^ AT1R, AT2R and prorenin receptors are expressed in all brain regions, though they highly expressed in microglia, astrocytes, neurons of cerebral cortex, hippocampus and basal ganglia.^32^ In addition, AT2R is less expressed in the brain compared to the AT1R.^38^ The expression of ACE is mainly in the astrocytes and neurons chiefly in the choroid plexus and cerebral vascular endothelium; however, ACE2 is primarily expressed in the brain stem to control the central mechanism of systemic blood pressure.^39^, ^40^

Brain AngII‐induced inflammation and oxidative stress is mediated by the activation of MAPK/JNK signalling pathway and NADPH oxidase, respectively.^37^, ^41^ AngII/AT1R complex is implicated in the development of neurotoxicity through activation of NMDA receptor and exaggeration of glutamatergic neurotransmission.^41^, ^42^ Moreover, brain AngII is further cleaved by aminopeptidase to generate AngIII and AngIV. AngIII has a neurotoxic effect through activation of AT1R, though AngIV has a neuroprotective effect through activation of AT4R.^37^, ^43^ Thus, AngII/AT1R/AngII/AT1R axis has detrimental effect on the brain through induction of inflammation, oxidative stress and neurotoxicity.

**FIGURE 2:**
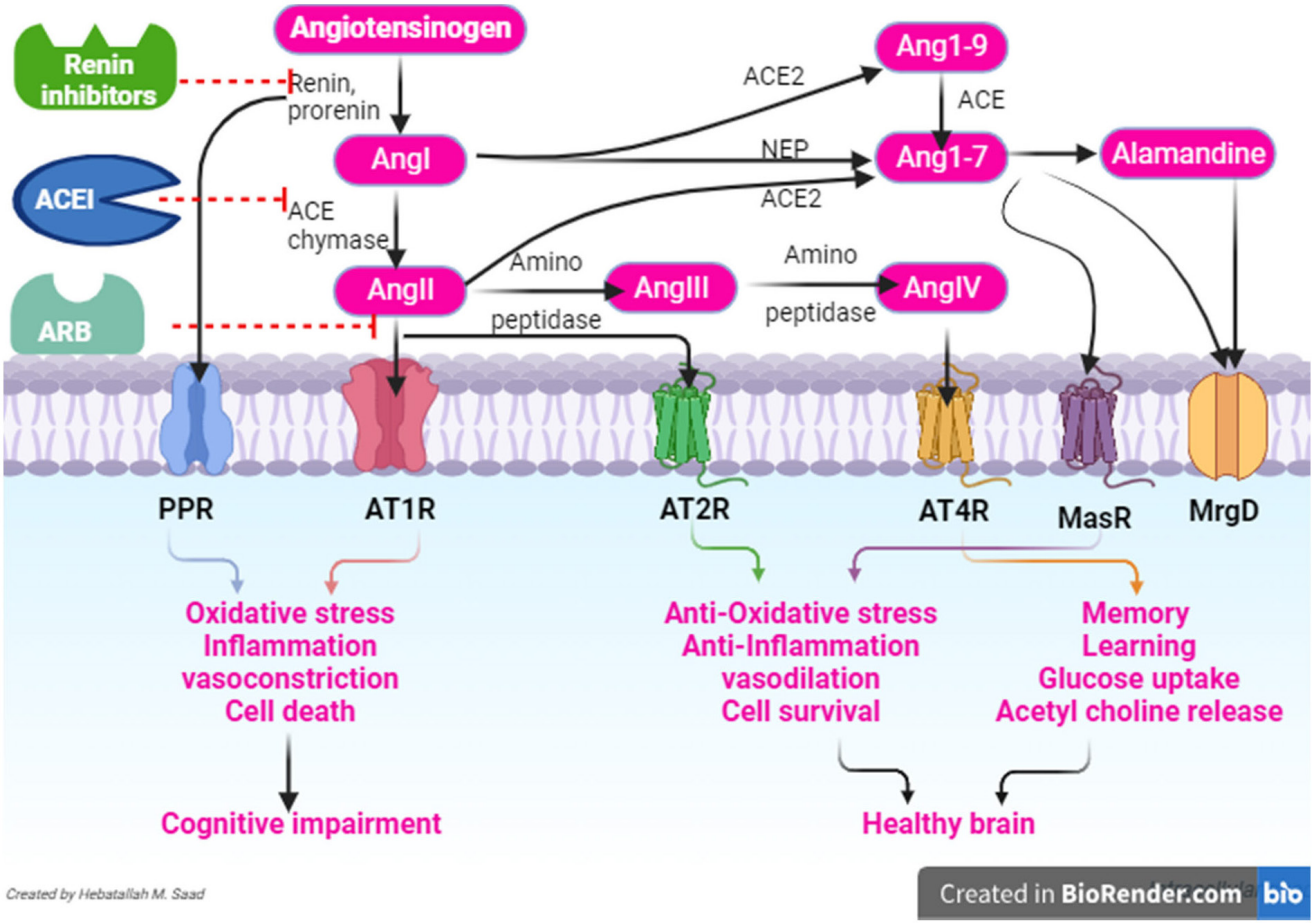
Role of brain RAS in healthy and diseased brain.

On the contrary, AngII/AT2R signalling pathway produces a neuroprotective effect by modulating the release of nitric oxide, and via activation of phospholipase A2, and can induce the release of arachidonic acid.^44^ AT2R inhibits the phosphorylation of epidermal growth factor and insulin receptors.^44^ In addition, AT1R blockade promotes the release of NO which is blocked by AT2R blockade proposing that AT2R is mainly involved in the synthesis and release of NO directly, or indirectly through the bradykinin pathway.^45^ In addition, AT2R promotes the expression of the neuroprotective sirtuin 1 (SIRT1).^46^ Besides, ACE2 triggers the conversion of AngII to the Ang1‐7 which has a neuroprotective effect by activating Mas receptor (MasR) which has antioxidant and anti‐inflammatory effect.^47^ Furthermore, Ang1‐7 via ACE2 is converted to the neuroprotective alamandine which activate Mas‐related G‐protein coupled receptor member D (MrgD).^48^ Therefore, ACE2/Ang1‐7/MasR and alamandine/MrgD pathways have neuroprotective effect, whereas; ACEAngII/AT1R pathway has a neurotoxic effect (Figure 2).

## 4 ROLE OF BRAIN RAS IN NEURODEGENERATIVE DISEASES

It has been reported that exaggerated of brain RAS is linked with the development and progression of different neurodegenerative diseases such as Alzheimer diseases (AD), amyotrophic lateral sclerosis (ALS), multiple sclerosis (MS) and other neurological disorders including traumatic brain injury and stroke.^49^

AD is a progressive neurodegenerative disease characterized by cognitive and memory impairment.^50^, ^51^, ^52^ AD is the commonest neurodegenerative disease in elderly and represents two third of all dementia cases. AD neuropathology is developed due to extracellular accumulation of neurotoxic amyloid beta (Aβ) and intracellular deposition of hyperphosphorylated tau protein which form neurofibrillary tangles (NFTs).^53^, ^54^ It has been shown that the over‐activation of brain RAS is associated with the development of cognitive impairment and memory deficits.^55^ It has been illustrated that overactivity of RAS in hypertensive patients triggers the development and progression of AD by activating of neuronal AT1R which induce oxidative stress and neuroinflammation.^55^ AT1R promotes the accumulation of Aβ and NFTs.^55^ Therefore, using of antihypertensive drugs mainly RAS inhibitors have recently shown to reduce AD risk.^56^ It has been observed that ARBs and ACEIs have potential neuroprotective role against AD pathogenesis through suppression of oxidative stress and inflammation‐induced brain neuronal injury.^57^ Torika et al.^58^ found that ACEI captopril inhibits microglia and attenuate neuroinflammation in AD mouse model. Excitingly, in vivo trans‐nasal administration of capotopril or perindopril reduce Aβ burden in transgenic mice,^58^ suggesting that intranasal delivery of ACEIs may be more effective than systemic administration of ACEIs in reducing Aβ burden and AD neuropathology. Therefore, RAS is concerned in the pathogenesis of AD and targeting of this system by ACEIs and ARBs could be effective in reducing AD even in non‐hypertensive patients.

Furthermore, ALS which is a third common neurodegenerative disease is characterized by degeneration of the motor neurons in the brain and spinal. Of note, 90% ALS cases are sporadic, and the remainder is familial type.^59^ It has been reported that CSF AngII which activate the neuroprotective AT2R is reduced in ALS patients compared to the healthy controls.^59^ Findings from preclinical studies highlighted that neuronal ACE activity is augmented in mice, and use of ACEI perindopril was shown to inhibit hippocampal microglia and oxidative stress in ALS mouse model.^60^ In addition, a case–control study observed that ACEIs reduce ALS risk by 57%^60^ suggesting that brain RAS plays a critical role in the pathogenesis of ALS, and use of ACEIs might be a protective strategy against ALS.

MS is a demyelinating disease of white matter in the CNS due to abnormal autoimmune response against myelin basic protein of oligodendrocytes.^61^ Development of peripheral autoreactive T and B immune cells together with derangement of BBB triggers this interaction result in demyelinating process in the white matter.^62^ In addition, progressive neuroinflammation and neuronal oxidative stress can induce neurodegeneration of grey matter leading to cognitive impairment and motor dysfunction.^63^, ^64^ In MS neuropathology, both of AT1R and AT2R are activated in the microglia result in pro‐inflammatory and anti‐inflammatory responses, respectively. Activated AT1R can induce demyelination while; AT2R promotes remyelination.^65^ Interestingly, AT1R activates Th1 immune response leading to activation of autoimmunity in MS. As well, ACE2 is downregulated while ACE is upregulated in MS patients.^66^ A case–control study performed by Haarmann et al.^67^ observed that CSF ACE activity is reduced in MS compared to healthy controls that might due to degeneration of perivascular astrocytes with impairment of ACE release into the CSF.^67^ Inhibition of brain AT1R and ACE by lisinopril and candesartan attenuate MS neuropathology through inhibition of autoimmune response.^68^ Therefore, dysregulation of brain RAS is implicated in the pathogenesis of MS.

These findings highlighted that brain RAS is intricate in the pathogenesis of neurodegenerative diseases, and modulation of brain RAS by ARBs or ACEIs may attenuate progression of these neurological disorders.

## 5 ROLE OF RAS IN PD

The association between PD and brain RAS was initially described by Allen et al^69^ They recognized that RAS is expressed in the SN pars compacta neighbouring dopamine‐containing cell bodies. As well, ATRs were identified in the presynaptic area suggesting that ARBs and ACEIs may affect dopamine synthesis and release.^69^ Moreover, evidence from preclinical and clinical studies supports the association between brain RAS and PD neuropathology.

### 5.1 Preclinical evidence

Different preclinical studies confirmed that dysregulation of brain RAS is associated with progressive degeneration of dopaminergic neurons in the SN.^70^, ^71^ Of note, dopaminergic neurons in the SN expressing AT1R more than AT2R^70^ thus; these neurons are highly susceptible the neurotoxic effects of AngII through induction of inflammation and oxidative stress. A postmortem study on human brain tissues of PD patients demonstrated that expression of AT2R was reduced in the SN due to loss of dopaminergic neurons.^72^ Therefore, activation of neuroprotective AT2R and inhibition of neurotoxic AT1R could a therapeutic strategy in the management of PD. It has been shown that ARB losartan protects dopaminergic neurons from the effect of 1‐methyl‐4‐phenyl‐1,2,3,6‐tetrahydropyridine (MPTP), and reduces the loss of dopaminergic neurons by about 72% in mice.^70^ In vitro study demonstrated that losartan attenuates rotenone‐induced injury and loss of dopaminergic neurons.^73^ Of note, AngII promotes the expression of tyrosine hydroxylase (TH) and dopamine biosynthesis leading to the formation of dopamine quinines which induce oxidative stress and injury of dopaminergic neurons in the SN.^74^ However, increasing the concentration of dopamine prevents aggregation of α‐Syn, but depletion of dopamine does not attenuate MPTP‐induced neurotoxicity.^75^, ^76^ Reduction of AngII by ACEIs but not by ARBs can reduce the neuroprotective effects of the AngII/AT2R axis and AngIV.^77^ Recent preclinical studies demonstrated that ARB candesartan inhibits exaggerated RAS activity in metabolic syndrome. Captopril, perindopril and losartan had proved greater neuroprotective role as evidenced by the increased serotonin, dopamine and acetylcholine levels in rotenone and MPTP models, and improve the non‐motor symptoms of PD.^78^

Indeed, expression of AT1R is increased while the expression of AT2R is reduced in aged brain, leading to augmentation of neurotoxic AngII/AT1R and reduction of neuroprotective AngII/AT2R with subsequent neurodegeneration.^71^ Increasing of AngII/AT1R axis promotes neuroinflammation and oxidative stress by inducing the expression of pro‐inflammatory cytokines and ROS, respectively.^71^

It has been shown that AngII/AT1R is highly expressed in the presynaptic neurons in the SN that mediate the release of dopamine. However, exaggerated AngII/AT1R in the SN promotes dopaminergic neurodegeneration through the activation of microglia and the development of neuroinflammation.^79^ In fact, exaggerated AngII/AT1R alone does not cause dopaminergic neurodegeneration unless activation of microglia^80^ signifying that AngII/AT1R‐induced microglial activation and neuroinflammation is the main pathway in the development of PD. Interestingly, exaggeration of ACE activity in the SN increases their susceptibility for oxidative stress, inflammation and progressive neurodegeneration and the development of PD.^81^ Therefore, ACEIs and ARBs are effective against motor dysfunction and cognitive impairment in PD.^23^, ^46^ Furthermore, the diminution of dopaminergic neurotransmission exacerbates neuroinflammation and dopaminergic neurodegeneration by upregulating AT1R.^38^ These observations proposed that the exaggerated AngII/AT1R axis is associated with the progressive degeneration of dopaminergic neurons in the SN and the development of PD.

On the contrary, the non‐classical RAS pathway produces a protective effect against the degeneration of dopaminergic neurons by opposing the effect of AngII/AT1R.^79^ In particular, the Ang1‐7/MASR axis which has a neuroprotective effect is highly reduced during ageing and predisposes to the development and progression of PD.^79^ Findings from animal studies indicated that the reduction of the Ang1‐7/MASR axis is linked with the degeneration of dopaminergic neurons in the SN.^79^ In addition, the upregulation of ACE2 by ARBs and ACEIs reduces the loss of dopaminergic neurons in the SN.^82^ Interestingly, RAS blockade decreases neuronal injury and mitigates motor and other features of PD.^83^ It has been shown that ACEI candesartan decreases the vulnerability to neurotoxic effect of 6‐OHDA through inhibition the expression and the release of pro‐inflammatory cytokines and NADPH oxidase.^84^ Increasing the expression of neuronal NADPH oxidase and dysregulation of AT1R/AT2R axis induce a progression degeneration of the dopaminergic neurons in the experimental aged rats.^84^ However, candesartan reduces the degeneration of the dopaminergic neurons in the SN of aged rats.^85^ Deletion of AT2R induces motor dysregulation and behavioural abnormality with the development of oxidative stress and neuroinflammation in mice.^38^

These preclinical findings highlighted that abnormal expression of brain RAS is implicated in PD neuropathology, and inhibition of AT1R or ACE by ARBs and ACEIs, respectively, can attenuate the pathogenesis of PD.

### 5.2 Clinical evidence

Importantly, exaggerated brain RAS is related with dysregulation of motor and non‐motor symptoms in PD patients.^23^ A longitudinal study included 107 PD patients with hypertension treated by ARBs or ACEIs followed for 10 years, showed that RAS inhibitors improve cognitive function and visuospatial memory in PD patients.^86^ Furthermore, long‐term use of RAS inhibitors attenuates the pathogenesis of PD and other neurodegenerative diseases.^87^ A nationwide cohort study from the Korean population involved RAS inhibitor users and non‐user patients revealed that patients RAS inhibitor users had low PD risk compared to non‐user patients.^86^

This finding suggests that ARBs are more effectual than ACEIs and other antihypertensive agents in mitigating cognitive deficits in PD. A cohort study included 90 PD patients (47 men and 43 women) showed that serum levels of AngI, AngII and Ang1‐7 are correlated with depressive symptoms in PD patients.^88^ A retrospective cohort study involved 107,207 hypertensive patients on different doses of ARBs followed for a median 8.4 years revealed that 1.1% of patients develop PD compared to 2.2% not treated by ARBs.^88^ Therefore, ARBs seem to have neuroprotective effects against the development of PD. In a nationwide cohort study on 62,228 patients with ischemic heart disease on ARBs followed for 10 years, and only 1086 patients developed PD.^89^ Of note, only ARBs with activity to penetrating BBB such as candesartan and telmisartan can reduce PD risk.^89^ Therefore, ARBs with higher penetrating activity through BBB have a higher efficacy against the development of PD.

Moreover, polymorphism of the *ACE* gene increases the risk for the development of PD.^90^ A case–control study on 127 PD patients and 198 healthy controls showed that homozygote *DD* genotype of *ACE* gene was higher in PD patients compared to healthy controls.^90^ However, a cohort study on 256 PD patients showed no significant association between single nucleotide polymorphisms (SNPs) of RAS components with PD risk and cognitive impairment.^91^ Though, these SNPs of RAS may affect the response and therapeutic efficacy of ACEIs and ARBs in PD patients. A meta‐analysis establish no significant connotation between *ACE* gene PD risks.^92^ It has been stated that ARBs are more effective in reducing PD risk than ACEIs owing to their selective inhibition of AT1R and increasing the neuroprotective ACE2/Ang1‐7/MASR axis.^93^ In addition, the higher concentration of AngII due to the effect of ARBs is further converted to AngIV which has a neuroprotective effect.^93^ In addition, ARBs promote the expression of proliferator‐activated receptor gamma‐associated peroxisome (PPAR‐γ) which has a neuroprotective effect.^94^ Notoriously, the neuroprotective effect of ARBs against PD may be sex‐dependent, as they produced more protective effects in women than men due to hormonal changes that affect expression of AT1R and AT2R.^95^

Of note, ACE2 has a momentous neuroprotective role against the pathogenesis of PD.^88^ Activation of ACE2 inhibits microglia activity thereby attenuate inflammatory and oxidative stress in neurodegenerative diseases.^88^ In a recent time, COVID‐19 pandemic which caused by SARS‐CoV‐2 virus is commonly related with downregulation of neuronal ACE2 results in the development of neuropsychiatric disorders.^96^ SARS‐CoV‐2‐induced downregulation in the dopaminergic neurons of the SN increases incidence of PD in COVID‐19 era.^96^ COVID‐19‐induced ACE2 downregulation is regarded the mechanistic pathway for the development of PD by inducing mitochondrial dysfunction, oxidative stress, impairment of BBB integrity and alteration of gut–brain axis.^96^ Al‐kuraishy et al.^1^ proposed that SARS‐CoV‐2‐induced neuroinflammation promotes PD neuropathology through induction the degeneration of the dopaminergic neurons in the SN.

Indeed, PD patients are often linked with other co‐morbidities such as diabetes mellitus, hypertension, dyslipidemia and inflammatory bowel diseases. These comorbidities adversely affect PD neuropathology by increasing neuroinflammation and accumulation of α‐Syn in the SN.^6^, ^17^, ^97^ It has been suggested that using of insulin sensitizing agent metformin in type 2 diabetic (T2D) patients and cholesterol lowering agents statins in patients with dyslipidemia and cardiovascular diseases reduce PD risk.^78^ Notably, RAS is exaggerated in hypertension, obesity, T2D, dyslipidemia and inflammatory bowel diseases.^98^, ^99^ Therefore, targeting of RAS by using ARBs or ACEIs may attenuate the detrimental effects of overactivated RAS in PD‐associated comorbidities. Regarding safety and efficacy of ARBs and ACEIs, different studies indicated that these agents are safe in PD patients, and did not interacts with other anti‐PD medications.^100^, ^101^

Taken together, these preclinical and clinical studies confirmed that exaggerated brain RAS is associated with PD neuropathology through different mechanisms that affect the viability of dopaminergic neurons.

## 6 MECHANISMS OF RAS‐INDUCED PARKINSON'S DISEASE

### 6.1 Neuroinflammation

**FIGURE 3:**
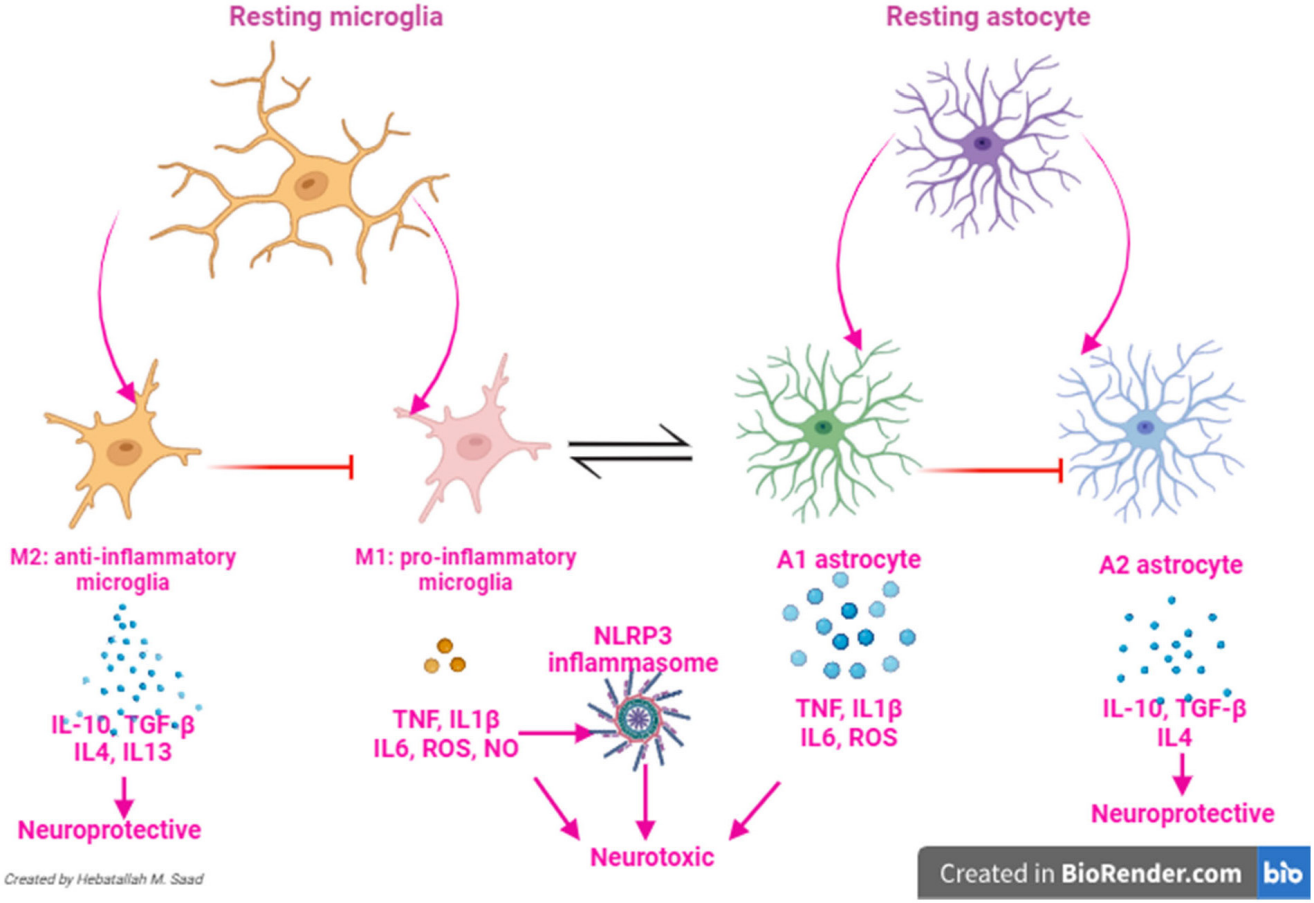
Differential effects of astrocytes and microglia.

One of most important defence mechanism of brain against exogenous pathogens, internal stimuli and neurodegeneration is the development of acute neuroinflammation to limit neuronal deaths in the CNS. Notably, astrocytes and microglia are involved in the development of acute neuroinflammation.^102^ However, in the chronic neuroinflammation, in addition to the activated astrocytes and microglia, peripheral immune and inflammatory cells can pass into the brain through injured BBB. Progressive chronic neuroinflammation triggers more neurodegeneration in the neurodegenerative diseases including PD, and the development of cognitive impairment by inhibiting synaptic plasticity and exacerbating of neuronal apoptosis.^103^ It has been established that exaggerated of peripheral RAS mainly AngII/AT1R axis through activation of T cells and immune cells induce the release of pro‐inflammatory cytokines which can reach through damaged BBB, and contribute in the neurodegenerative process.^103^, ^104^ Conversely, the non‐classical pathway ACE2/Ang1‐7/MasR through counteracting the inflammatory role of the classical axis can inhibits the progression of neuroinflammation.^103^, ^104^ Importantly, the central AngII plays an important role in the development and progression of neuroinflammation by upregulating neuronal NADPH oxidase.^104^ Besides, uses of the ARBs and ACEIs can decrease the progression of neuroinflammation by inhibiting the expression of pro‐inflammatory cytokines.^105^ A preclinical study found that ARB losartan suppresses LPS‐induced oxidative stress and inflammation and the development of neuroinflammation.^106^ Similarly, candesartan attenuates the development of neuroinflammation by improving brain insulin signalling which inhibit oxidative stress and neuroinflammation in the experimental rats.^106^ In addition, a low‐dose of candesartan enhances the expression of anti‐inflammatory cytokines such as IL‐10 by upregulating the anti‐inflammatory AT2R.^107^, ^108^ Thus, ACEIs and ARBs could be effective against the pathogenesis of PD by inhibiting the development of chronic neuroinflammation.^109^ It has been established by different preclinical studies that neuroinflammation is highly implicated in the pathogenesis of PD through induction the degeneration of the dopaminergic neurons in the SN.^109^, ^110^ In neuroinflammation, dysregulation of microglial activation triggers the progression of PD neuropathology. Notably, microglia type 1 has a neurotoxic effect by inducing the expression and the release of pro‐inflammatory cytokines, though microglia type 2 has a neuroprotective role by inducing the release of anti‐inflammatory cytokines.^109^, ^110^ A similar effect observed by type 1 and type 2 astrocytes^109^, ^110^ (Figure 3).

Remarkably, resting microglia in the SN represent 12% of cells in this region.^111^ Expression of microglia type 1 in cooperation with A1 astrocyte promotes neuroinflammation in PD.^111^ Importantly, neuroinflammation in the early phase of PD neuropathology enhances the elimination of α‐Syn, though in advance PD neuropathology neuroinflammation induces progressive neurodegeneration in PD through activation of Toll‐like receptor 4 (TLR4).^112^ TLR4 is regarded as a critical receptor involved in PD neuropathology.^112^ A clinical study revealed that levels of pro‐inflammatory cytokines are in the CSF and plasma are augmented in PD patients.^113^ Microgliosis which is a local proliferation of microglia triggers neurodegeneration in PD by inducing neuroinflammation.^114^ Biomarkers of neuroinflammation such as TNF‐α and IL‐1β are increased in PD patients.^115^ Furthermore, aggregation of α‐Syn promotes neuroinflammation through the induction of different inflammatory singnaling pathways such as NLRP3 inflammasome and NF‐κB.^116^ These verdicts indicated that dysregulated RAS can induce PD, and the use of ARBs or ACEIs can mitigate PD neuropathology by inhibiting neuroinflammation.

### 6.2 Brain RAS and inflammatory signalling pathways

#### 6.2.1 NF‐κB

NF‐κB is one of most important inflammatory signalling pathway involved in various physiological and pathological disorders.^117^ Of note, two important NF‐κB pathways are identified including conical and non‐conical that are differed in their response to the pathological stimuli. The conical NF‐κB pathway is triggered in response to the exogenous stimuli through modulation of IκB and the release of NF‐κB which via activation of the negative regulator IκB suppress further release of activated NF‐κB.^118^, ^119^ Conversely, the non‐conical NF‐κB pathway which involved in the regulation of immune response is activated by endogenous signalling such as TNF‐α via stimulation of NF‐κB inducing kinase.^118^, ^120^ The normal NF‐κB signalling regulates the survival of the dopaminergic neurons of the SN.^121^ However, dysregulation of NF‐κB signalling pathway is intricate in PD neuropathology through induction the development and progression of neuroinflammation and direct neurodegeneration in the SN.^121^ Particularly, misfolded and mutant α‐Syn from injured dopaminergic neurons triggers the release of the NF‐κB from activated microglia.^121^ Furthermore, exaggeration of NF‐κB signalling is associated with dysregulated brain RAS.^122^ Interestingly, AngII via the expression of TLR4 activates NF‐κB signalling leading to the induction of pro‐inflammatory and fibrotic effects. As well, myeloid differentiation 2 (MD2) molecules through activation of TLR4 also mediates the pro‐inflammatory effect of AngII.^122^ Thus, activated MD2/TLR4 complex is necessary for the pro‐inflammatory effect of AngII. In addition, AngII through the activation of brain TLR4 may induce neurodegeneration in the SN.^123^ Importantly, TLR4 mediates the effect of MPTP‐induced injury of the nigrostriatal pathway.^124^ Supporting to the finding, genetic deletion of neuronal TLR4 in the SN attenuates AngII‐induced neurodegeneration in the SN.^122^

However, ACE2/Ang1‐7/MASR axis inhibits NF‐κB expression in the 6‐OHDA PD animal model by inhibiting NADPH oxidase.^125^ Rabie et al.,^126^ illustrated that Ang1‐7 via activation of MASR improves motor dysregulation in PD by inhibiting NF‐κB expression. It has been revealed that ARB candesartan attenuates LPS‐induced cognitive impairment in mice by inhibiting NF‐κB expression.^127^ These verdicts indicated that AngII/AT1R is involved in the PD neuropathology, and inhibition of this pathway by ARBs can mitigate PD neuropathology via suppression of NF‐κB signalling.

#### 6.2.2 NLRP3 inflammasome

NLRP3 inflammasome is an important inflammatory signalling pathway intricate in the regulation of innate immune response through activation the expression and release of IL‐1β and caspase‐1.^128^ NLRP3 inflammasome is often stimulated by the ROS and NF‐κB signalling. Preclinical evidences illustrated that exaggerated NLRP3 inflammasome is implicated in the pathogenesis of PD by inducing the development of neuroinflammation and triggering progressive apoptosis of the dopaminergic neurons within the SN.^129^

Notably, exaggerated NLRP3 inflammasome signalling triggers misfolding and the accumulation of α‐Syn which also triggers stimulation of NLRP3 inflammasome.^130^ A case–control study observed that plasma NLRP3 inflammasome level is increased in PD compared to controls,^130^ and NLRP3 inflammasome level in both plasma and the CSF are correlated with CSF α‐Syn.^131^ These findings suggest that NLRP3 inflammasome activity is highly involved in PD neuropathology.

On the contrary, the classical AngII/AT1R axis encourages the activation of NLRP3 inflammasome.^132^ A preclinical study conducted by Zhao et al.^132^ established that NLRP3 inflammasome mediates the effect of AngII‐induced acute kidney injury in mice. Supporting this idea, NLRP3 inflammasome knockout mice are resistant to AngII‐induced podocyte injury.^132^ An in vitro study demonstrated that AngII promotes the expression of NLRP3 inflammasome via the release of Ca^2+^ in cardiac fibroblast.^133^ Therefore, ARB losartan can prevent the activation of NLRP3 inflammasome in cardiac fibroblast.^133^ Interestingly, upregulation of NLRP3 inflammasome is more evident in aged and PD model mice.^134^ Furthermore, the expression of NLRP3 inflammasome in the SN is increased in the experimental PD model and contributes in the neurodegenerative process. Besides, the administration of AngII promotes the activation of NLRP3 inflammasome and degeneration of dopaminergic neurons in the SN in rats with 6‐OHDHA‐induced PD.^134^ Furthermore, ARB candesartan attenuated NLRP3 inflammasome signalling and loss of dopaminergic neurons in the SN.^134^ The neuroprotective effects of ARBs against activation of NLRP3 inflammasome is not only mediated by suppression of AngII/AT1R but may be through stimulation of ACE2/Ang1‐7/MASR axis which inhibits NLRP3 inflammasome.^135^ Duan and his colleagues revealed that Ang1‐7 analogue AVE0991 prevents astrocyte‐induced neuroinflammation by inhibiting the expression of NLRP3 inflammasome.^42^ Likewise, the Ang1‐7/MASR axis reduces microglia‐induced neuroinflammation via inhibition of NLRP3 inflammasome.^42^

These findings proposed that the over‐activated classical AngII/AT1R axis and downregulated Ang1‐7/MASR axis in the SN are associated with PD neuropathology by persuading the expression of NLRP3 inflammasome. Besides, inhibition of the AngII/AT1R axis and stimulation of the Ang1‐7/MASR axis in the SN by ARBs can mitigate PD neuropathology.

#### 6.2.3 RAS and oxidative stress

Of note, ROS are formed continually generated by the body tissues, and are neutralized and eliminated by body antioxidant system.^136^ Excessive generation and/or impairment of endogenous antioxidant capacity promote the development of oxidative stress.^137^ Indeed, the mitochondria the main site for the generation of ROS during ATP production.^137^ It has been shown that the brain is more vulnerable to neurotoxic effect of oxidative stress than other organs because of advanced metabolic activity and low antioxidant system.^138^ In addition, oxidative stress is regarded as a central mechanism intricate in the development of neurodegenerative diseases including PD.^139^ Moreover, exaggeration of RAS as in obesity, diabetes and hypertension is associated with the development of oxidative stress and mitochondrial dysfunction.^136^ In addition, exaggeration of RAS through induction of oxidative stress and mitochondrial dysfunction can cause skeletal muscle injury in mice.^140^ Likewise, AngII‐induced endothelial dysfunction and vascular injury is mainly mediated through the induction of oxidative stress and mitochondrial dysfunction.^141^ As well, AngII‐induced cognitive dysfunction and neurodegeneration is mainly mediated through induction of oxidative stress and mitochondrial dysfunction.^142^ The neuropathological role of AngII is related to the inhibition the expression of *ACE2* gene with subsequent inhibition of brain non‐classical pathway.^125^, ^143^ Conversely, Ang1‐7 attenuates AngII‐induced cerebral endothelial dysfunction through suppression of brain NADPH oxidase.^144^ These findings indicated a potential interaction between the classical and non‐classical pathways in the brain. Thus, activation of the non‐classical pathway and inhibition of the classical pathway could be a potential mechanistic pathway to prevent the development of PD and other neurodegenerative diseases. Supporting this claim, Ketan et al.,^145^ illustrated that ARBs can attenuate the progression of oxidative stress and mitochondrial dysfunction. Likewise, Gupta et al.^146^ established that ARB azilsartan attenuates experimental stroke in rats by inhibiting mitochondrial dysfunction, oxidative stress and associated neuroinflammation.

In PD neuropathology, mitochondrial dysfunction and related oxidative stress augments the neurodegeneration in the SN.^147^ In MPTP and rotenone‐induced PD, oxidative stress and mitochondrial dysfunction are highly augmented causing neurodegeneration in the SN.^148^ Interestingly, oxidative stress and mitochondrial dysfunction are developed in the early phase of PD neuropathology. Impairment of mitochondrial electron transport and alteration of mitochondrial biology might be the principal events in the pathogenesis of PD.^149^ Furthermore, mitochondrial dysfunction and related oxidative stress promote the aggregation of α‐Syn in the dopaminergic neurons of the SN.^150^

In sum, AngII‐induced mitochondrial dysfunction and oxidative stress might be the main mechanism in the development of PD. Suppression of brain AngII/AT1R axis by highly penetrating ARBs not only reduce the deleterious effect of this axis, but also upregulate the neuroprotective non‐classical pathway which mitigate PD neuropathology.

#### 6.2.4 Brain RAS and dysregulation of BDNF

The neurtophic and neuromodulator brain derived neurotrophic factor (BDNF) plays a critical role in the modulation of synaptic plasticity, memory and cognitive function. BDNF is a member of brain neurotrophin family involved in neurogenesis and modulation of cognitive function.^151^ BDNF signalling is mediated mainly through activation of tyrosine kinase receptor B (TrkB) and to lesser extent through p75NT which is the primary receptor of pro‐BDNF.^151^, ^152^ It has been observed that CNS BDNF is mainly released from the hippocampus and hypothalamus. In addition, BDNF is also released from different tissues and platelets. The peripheral BDNF cannot crosses the BBB, indicating that brain BDNF has different physiological effect than the peripheral one.^151^, ^152^ Different studies illustrated that BDNF signalling is dysregulated in PD patients compared to healthy controls. BDNF level is decreased in the early stage of PD due to neurodegenerative process, though in advanced PD the BDNF level is increased as a compensatory mechanism to alleviates inflammatory and oxidative stress disorders.^153^ Chang et al.^154^ established that activation of BDNF signalling reduces motor deficit and cognitive dysfunction in mouse PD model. Improvement of BDNF signalling by antidepressants promotes cognitive and motor functions in PD patients.^155^ Remarkably, brain RAS regulates the expression of BDNF signalling, though exaggerated of brain RAS inhibits BDNF signalling mainly BDNF/TrkB via TLR4‐dependent mechanism.^127^ many preclinical studies demonstrated that ARB candesartan promotes BDNF/TrkB in the astrocytes.^156^ In addition, candesartan attenuates LPS‐induced cognitive impairment by restoring the activity of brain BDNF/TrkB in mice.^127^ Likewise, ARB valsartan improves neurogenesis in mice via BDNF/TrkB‐dependent pathway.^157^ As well, ACEI captopril attenuates cognitive impairment in the experimental rats through augmentation of brain BDNF/TrkB signalling.^158^ On the other side, the non‐classical pathway of brain RAS primarily Ang1‐7/MasR had been shown to improves the cognitive in the animal model studies by increasing of brain BDNF/TrkB signalling.^99^, ^159^ Furthermore, AngIV improves memory and cognitive functions through activation of brain BDNF/TrkB signalling and inhibiting of brain oxidative stress in the hippocampus in an animal model study.^160^ These findings highlighted that dysregulation of brain RAS is associated with augmentation of PD neuropathology through inhibition of brain BDNF/TrkB signalling. Therefore, ARBs and ACEIs may improve the cognitive and reduce PD by restoring brain BDNF/TrkB signalling.

## 7 CONCLUSIONS AND PERSPECTIVES

PD is one of most common neurodegenerative disease of the brain related to the progressive accumulation of mutant and insoluble α‐Syn and Lewy bodies in the dopaminergic neurons of the SN. The brain RAS is involved in the regulation of memory and cognitive function, and in the controlling of central blood pressure. However, exaggerated brain RAS is implicated in the pathogenesis of different neurodegenerative diseases including PD. Notably, brain RAS has two important axes; the classical axis which mediated by AngII/AT1R has a detrimental effect and involved in the pathogenesis of PD. However, the non‐classical axis which mediated by ACE2/Ang1‐7/MasR and AngII/AT2R has a neuroprotective effect against the development of PD. Overactivity of AngII/AT1R and deregulation of ACE2/Ang1‐7/MasR and AngII/AT2R trigger progressive neurodegeneration in the SN and the development of PD. Targeting of brain RAS by ACEIs and ARBs result in the downregulation of the neurotoxic AngII/AT1R axis, and upregulation of the neuroprotective ACE2/Ang1‐7/MasR and AngII/AT2R. Moreover, ACEIs and ARBs through modulation of the brain RAS can prevent oxidative stress, mitochondrial dysfunction, neuroinflammation and dysregulation of BDNF/TrkB signalling which are involved in PD neuropathology.

Cotherapy of ACEIs or ARBs with anti‐PD medications such as L‐DOPA could be a novel therapeutic strategy in the management of PD in both hypertensive and non‐hypertensive patients. ACEIs and ARBs can improve survival of dopaminergic neurons in the SN through BDNF‐dependent pathway, thereby reducing motor dysfunction such as ON–OFF episodes which are induced by L‐DOPA. Furthermore, any patients with cardiometabolic disorders should encourage taking RAS inhibitors unless there is any contraindications to prevent the development and progression of PD. A special delivery system for ACEIs or ARBs such as trans‐nasal route may increase the efficacy of these agents in the management of PD, and reduce systemic adverse effects. These claims need preclinical and clinical trials to be verified in the management of PD.

## AUTHOR CONTRIBUTIONS

**Zainah Al‐Qahtani:** Writing – review and editing (equal). **Hayder M. Al‐kuraishy:** Conceptualization (equal); validation (equal). **Ali I. Al‐Gareeb:** Resources (equal); validation (equal); visualization (equal). **Ali K. Albuhadily:** Conceptualization (equal); resources (equal). **Naif H. Ali:** Writing – review and editing (equal). **Athanasios Alexiou:** Supervision (equal); visualization (equal); writing – original draft (equal). **Marios Papadakis:** Funding acquisition (equal); writing – original draft (equal). **Hebatallah M. Saad:** Writing – original draft (equal); writing – review and editing (equal). **Gaber El‐Saber Batiha:** Writing – review and editing (equal).

## FUNDING INFORMATION

This work was supported by the University of Witten‐Herdecke Germany.

## CONFLICT OF INTEREST STATEMENT

The authors declare no conflict of interest.

## Data Availability

Data sharing is not applicable to this article as no new data were created or analysed in this study.
